# Triazoloquinazolines as a new class of potent α-glucosidase inhibitors: *in vitro* evaluation and docking study

**DOI:** 10.1371/journal.pone.0220379

**Published:** 2019-08-14

**Authors:** Hatem A. Abuelizz, El Hassane Anouar, Rohaya Ahmad, Nor Izzati Iwana Nor Azman, Mohamed Marzouk, Rashad Al-Salahi

**Affiliations:** 1 Department of Pharmaceutical Chemistry, College of Pharmacy, King Saud University, Riyadh, Saudi Arabia; 2 Department of Chemistry, College of Sciences and Humanities, Prince Sattam bin Abdulaziz University, Al Kharj, Saudi Arabia; 3 Faculty of Applied Sciences, Universiti Teknologi MARA, shah Alam, Selangor Darul Ehsan, Malaysia; 4 Chemistry of Natural Products Group, Centre of Excellence for Advanced Sciences, National Research Centre, Dokki, Cairo, Egypt; Weizmann Institute of Science, ISRAEL

## Abstract

Previously, we synthesized triazoloquinazolines **1–14** and characterized their structure. In this study, we aimed to evaluate the *in vitro* activity of the targets **1–14** as α-glucosidase inhibitors using α-glucosidase enzyme from *Saccharomyces cerevisiae* type 1. Among the tested compounds, triazoloquinazolines **14**, **8**, **4**, **5**, and **3** showed the highest inhibitory activity (IC_50_ = 12.70 ± 1.87, 28.54 ± 1.22, 45.65 ± 4.28, 72.28 ± 4.67, and 83.87 ± 5.12 μM, respectively) in relation to that of acarbose (IC_50_ = 143.54 ± 2.08 μM) as a reference drug. Triazoloquinazolines were identified herein as a new class of potent α-glucosidase inhibitors. Molecular docking results envisaged the plausible binding interaction between the target triazoloquinazolines and α-glucosidase enzyme and indicated considerable interaction with the active site residues.

## Introduction

Diabetes mellitus (DM) is a life-threatening, chronic metabolic disorder of multiple etiologies, characterized by hyperglycemia accompanied with disturbances in protein, fat, and carbohydrate metabolism [1–4]. In 2011, approximately 366 million people were diagnosed with diabetes, according to the International Diabetes Federation (IDF) studies. This number is predicted to reach 522 million by 2030. Diabetes severely damages several vital organs, leading to heart attack, stroke, and neuropathy complications [5,6]. Diabetes has become pandemic in human society owing to its prevalence, and high rate of associated mortality and morbidity [7]. Thus, treating hyperglycemia and controlling the subsequent complications are the main aims of diabetes therapy.

According to a report by the Pharmaceutical Research and Manufacturers of America (PhRMA), 182 new antidiabetic agents (30 for type 1, 100 for type 2, and 52 for related condition of DM) have been developed by American biopharmaceutical companies, and these drugs are still either in clinical trials or under review by the Food and Drug Administration [7]. Currently, a wide range of oral antidiabetic drugs are being utilized due to the absence of efficient and affordable interventions [7]. In most cases, the prescribed antidiabetic drugs are responsible for various side effects such as liver problems, diarrhea, lactic acidosis, and high rate of secondary failure.

α-Glucosidase is present in the brush border membrane of the intestine. It catalyzes the hydrolysis of α-(1→4)-glycosidic linkage of sugar (disaccharides and starch), releasing free monosaccharides (α-D-glucose) during the final step of carbohydrate digestion. Thus, α-glucosidase inhibitors (AGIs) can suppress postprandial hyperglycemia and decrease carbohydrate digestion rate; therefore, reduce the glucose level in the blood stream [1,8–13].

α-Glucosidase inhibitors are a unique class of anti-diabetic drugs, described as an attractive therapeutic target for type-II diabetes and may also be used for other diseases in which carbohydrates or its biosynthesis are potential targets including cancer, hyperlipoproteinemia, HIV, and obesity [14,15]. In contrast to other oral antidiabetic drugs, AGIs exert their effect locally in the intestine, rather than modulating some biochemical processes in the body. Accordingly, extensive research has been carried out, elucidating the possible role of AGIs in the treatment of prediabetic conditions, such as impaired glucose tolerance IGT and impaired fasting glucose IFG [16].

α-Glucosidase inhibitors (acarbose, miglitol, and voglibose) are characterized by similar structure to that of the sugar moieties (disaccharides and oligosaccharides); thus, they bind to α-glucosidase via the carbohydrate site [17–19]. During the last two decades, the AGIs have been introduced as antidiabetic drugs and recommended as the first line therapy by the IDF and American Association of Clinical Endocrinologists (AACE) [20]. These drugs are well tolerated; however, their localized gastrointestinal adverse effects such as diarrhea, bloating, and flatulence restrict the long-term acquiescence for treatment [8,9].

**Fig 1:**
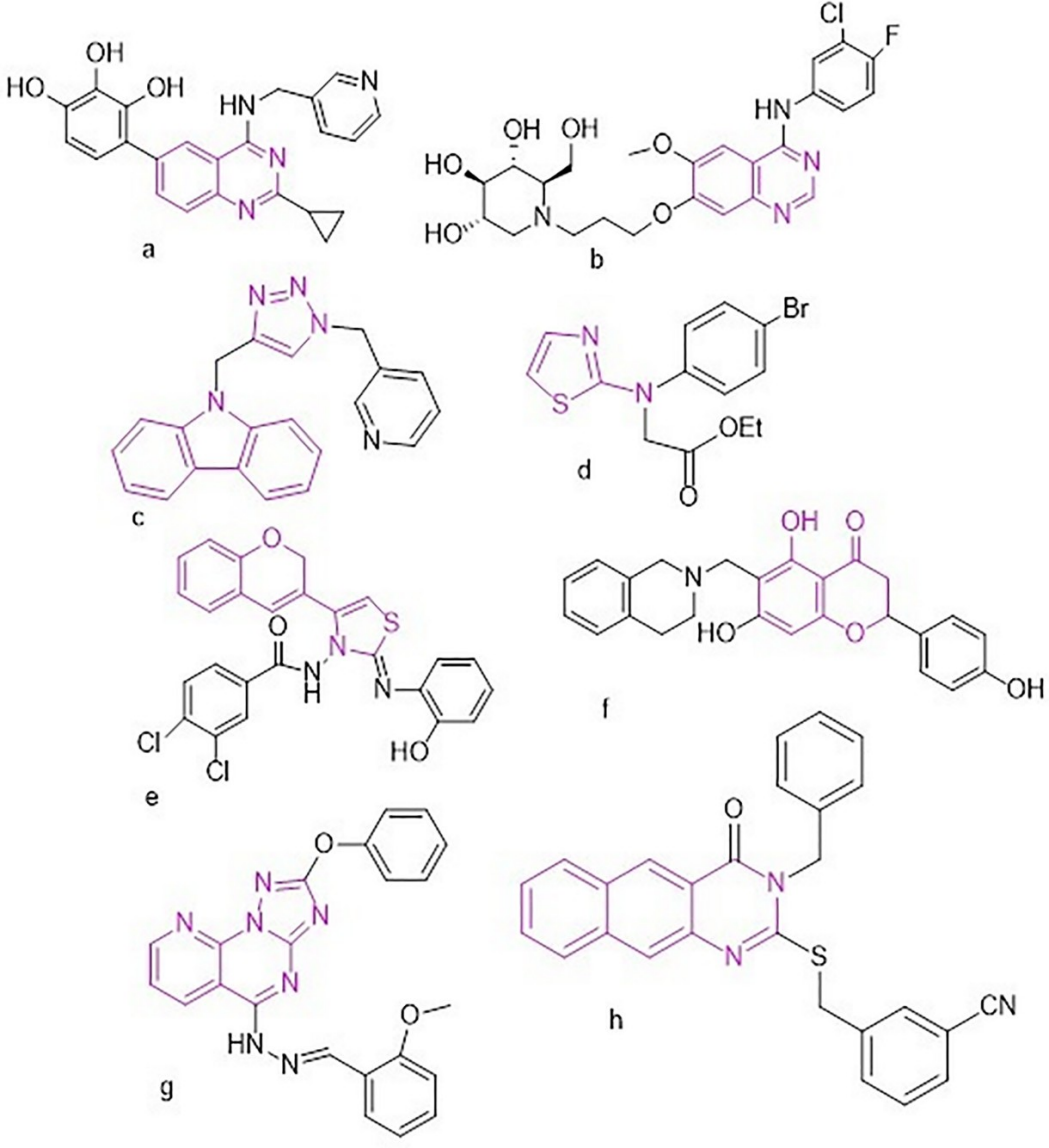
Synthetic routes for compounds 1–14.

Recently, nitrogen-containing heterocyclic compounds without glycosyl, such as triazole, quinazoline, imidazole, thiazole, and pyrazole, have been documented as potent *in vitro* AGIs. Moreover, some natural and synthesized flavonoids (such as luteolin, naringenin, anthocyanins, and baicalein), coumarin, chromones and their derivatives (Fig 1 d-f) have been targeted as potent AGIs [8,9,21–26]. Triazole and quinazoline derivatives have gained considerable attention owing to their important pharmacological activities (Fig 1). For instance, carbazole-linked triazole compounds have shown potent α-glucosidase inhibition activity in relation to that of the standard acarbose (Fig 1C) [22]. Furthermore, substituted quinazolines have been reported to possess antihyperglycemic and α-glucosidase inhibition effects (Fig 1A and 1B). In particular, the modified 4-Cl/Br-2-phenyl-quinazolines reversibly inhibited α-glucosidase enzyme activity in a non-competitive manner (Fig 1B), resulting in excellent inhibitory activity comparable to that of acarbose [24].

In our previous study, we have shown that benzoquinazoline derivatives have the potential to act as a new class of AGIs. Their IC_50_ value was 69.20 ± 1.76, 59.60 ± 0.52, 49.40 ± 0.50, 50.20 ± 0.37, and 88.20 ± 0.89 μM, whereas that of acarbose was 143.54 μM [8]. In addition, our 2-phenoxy-pyridotriazolopyrimidine was found to possess potent α-glucosidase inhibitiory (104.07 ± 4.89 μM) in relation to acarbose (143.54 μM) (Fig 1G) [9]. Particularly, our study suggested that different types of interactions between benzoquinazolines and α-glucosidase, such as H-bonding, free binding energy, closest amino acids, and hydrophobic effects, are key factors involved in increasing the inhibitory effects [8]. Further, structure–activity relationship (SAR) study on benzoquinazolines suggested that the type of functional groups attached at position 2 of benzoquinazoline was found to increase α-glucosidase inhibitory activity [8] and that the activity largely depends on electron donating/withdrawing substituents (Fig 1H).

The undesired effects of available AGIs have encouraged researchers to discover safer and new generation of α-glucosidase inhibitor agents. Therefore, it is necessary to clarify how the α-glucosidase inhibitory activity can be affected by combining the quinazoline and triazole moieties in one molecule. However, to the best of our knowledge, there are no studies on the relationship between triazoloquinazolines and α-glucosidase inhibition activity. To clarify and gain deeper insight into the SAR of triazoloquinazolines with α–glucosidase, in the present study, we evaluated the *in vitro* α-glucosidase inhibitory activity of the target triazoloquinazolines (**1–14**).

## Materials and methods

### α-Glucosidase inhibitory assay

#### Chemicals and reagents

Sodium phosphate monobasic, sodium chloride, 0.2g of potassium chloride and 0.2g potassium dihydrogen phosphate All chemical and reagent used were purchased from Sigma-Aldrich (St Louis, Missouri).

#### Preparation of reagents

For phosphate-buffered saline (PBS), NaH₂PO₄ (1.4 g), NaCl (8 g), KCl (0.2 g), and KH₂PO4 (0.2 g) were dissolved in deionized H_2_O (1 L) at 20°C. Using NaOH (1M) and HCl (1M), the pH of the solution was adjusted to 6.5 at 20°C. *p*-Nitrophenyl-α-D-glucopyranoside (2 mM) (*p*-NPG-substrate) was prepared by dissolving p-NPG (6 mg) in buffer solution (10 mL). α-Glucosidase enzyme Type 1, lyophilized powder (Sigma-Aldrich) from *Saccharomyces cerevisiae* (a mixture of MAL12 and MAL32) was employed. The enzyme solution was prepared by dissolving 1 mg of in 1000 μL of cold PBS (pH 6.5). Subsequently, 50 μL of α-glucosidase enzyme solution was mixed with cold buffered saline (12 mL) to a concentration of 0.125 unit/mL. For sample screening, a solution of 200 μg/mL concentration was prepared in 100% DMSO, and then subjected to two-fold serial dilution in 5% DMSO in a 96-well microplate to obtain concentrations of 100, 50, 25, 12.5, 6.25, 3.13, and 1.56 μg/mL. The samples were screened using Epoch 2 Microplate Spectrophotometer from BioTek. To each well of the 96-well microplate, 10 μL of DMSO (5%) was added. The stock solution (10 μL) was added to the first well and mixed. Then, 10 μL of sample was withdrawn from the first well and added to the second well, and the process was repeated for two-fold serial dilution. Subsequently, 20 μL of α-glucosidase enzyme, 40 μL of PBS (pH 6.5), and 20 μL of deionized H_2_O were added to each well of the 96-well microtiter plate with constant stirring. The plate was pre-incubated at 37°C for 10 min, and then 10 μL of p-NPG (2 mM) solution was added to the mixture; its absorbance at 0 min was measured at a wavelength of 405 nm. The reaction mixture was incubated at 37°C for 30 min, and subsequently, its absorbance was measured. Dimethyl sulfoxide (5%) was used as the negative control and acarbose (200 μg/mL)(Sigma-Aldrich) as the positive control. The experiment was performed in triplicate. According to a previously described methodology [27], the α-glucosidase inhibitory activity was determined and the inhibition (%) of this activity was calculated using the following equation:

$$\%inhibition=\frac{(A_{30min}-A_{0min})control-(A_{30min}-A_{0min})exp}{(A_{30min}-A_{0min})control}\times 100$$

#### Molecular docking

Autodock package was used to assess triazoloquinazolines as AGIs [28]. The original docked ligands were downloaded from the RCSB data bank website with PDB code 3W37 [28], and X-ray analysis was performed to coordinate the target α-glucosidase. Molecular docking was performed according to Abuelizz et al [8, 9, 29].

## Results and discussion

**Fig 2:**
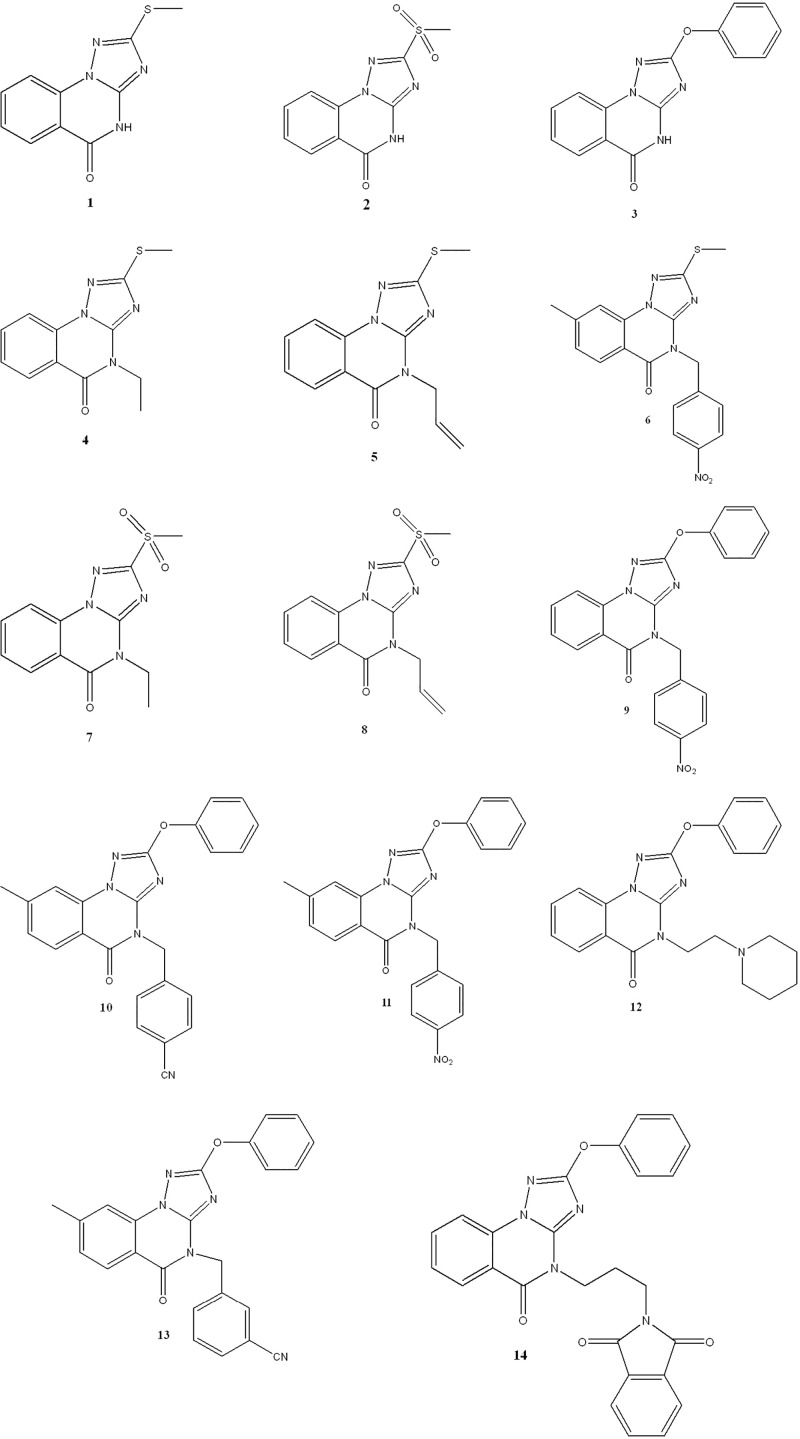
Structure of the target triazoloquinazolines (1–14).

**Fig 3:**
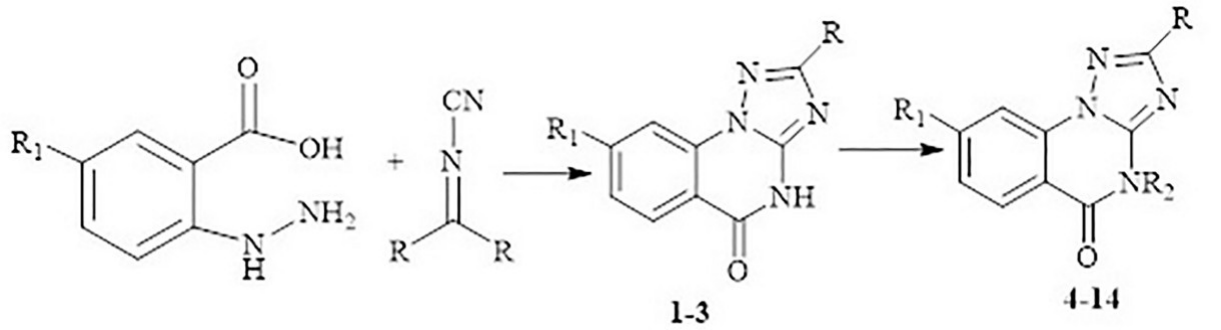
Synthetic routes for compounds 1–14.

The target triazoloquinazolines **1**–**14** (Fig 2) were characterized in our previous study [30–34]. Cyclocondensation reaction of dialkyl-*N*-cyanoimido(dithio)carbonates with 2-hydrazinobnezoic acid afforded the parents **1** and **3**, whereas the parent **2** was obtained by oxidation of **1** with H_2_O_2_ (Fig 3). Reaction of compounds **1–3** with appropriate alkyl(heteroalkyl)halides in basic medium furnished the *N*-alkylated products **4–14.**

In the present study, we assayed their enzymatic inhibitory activity against α-glucosidase. The IC_50_ value of the target molecules are listed in Table 1 and S1 Fig

**Table 1:** α-Glucosidase inhibitory activity of triazoloquinazolines 1–14.

Sample	IC_50_ (μM)
**1**	155.86 ± 1.36
**2**	180.34 ± 1.28
**3**	83.87 ± 5.12
**4**	45.65 ± 4.28
**5**	72.28 ± 4.67
**6**	16.24% inhibition at 0.28 μM
**7**	19.15% inhibition at 0.34 μM
**8**	28.54 ± 1.22
**9**	16.89% inhibition at 0.24 μM
**10**	14.83% inhibition at 0.25 μM
**11**	19.52% inhibition at 0.23 μM
**12**	97.53 ± 0.94
**13**	27.93% inhibition at 0.21 μM
**14**	12.70 ± 1.87
**Acarbose**	143.54 ± 2.08

Generally, the structural diversity of triazoloquinazolines based on substituent groups at positions 2 and 4 largely contribute to their diverse inhibitory activities.

Compounds **1**–**14** were investigated for their inhibitory potential against α-glucosidase, and they demonstrated significant activity with the IC_50_ values ranging between 12.70 ± 1.87 and 180.34 ± 1.28 μM (Table 1 and S1 Fig). Triazoloquinazolines **3**–**5**, **8**, **12,** and **14** were the most potent α-glucosidase inhibitors with the IC_50_ values of 83.87 ± 5.12, 45.65 ± 4.28, 72.28 ± 4.67, 28.54 ± 1.22, 97.53 ± 0,94, and 12.70 ± 1.87 μM, respectively, in relation to that of acarbose (IC_50_ = 143.54 μM). Triazoloquinazolines **1** and **2** showed good inhibitory effect (155.86 ± 1.36 and 180.43 ± 1.28 μM), which was comparable to that of the standard acarbose, whereas compounds **6**, **7**, **9**–**11,** and **13** showed less than 50% inhibition, and were not screened for their IC_50_ values. Triazoloquinazoline **14**, **8,** and **4** were more active, and approximately 12, 5, and 3 times more potent than acarbose, respectively. It is interesting to note that all the target compounds have the same triazoloquinazoline scaffold and differ only in the substituents at positions 2 and/or 4 with the ‘parent’ compounds **1**, **2** and **3** bearing a 2-SMe, 2-SO_2_Me or 2-OPh substituent and a free amine position 4. Therefore, the difference in their inhibitory potential might be attributed to the structural diversity of the different substituents designed at position 2 and/or 4-NH along with the presence of 8-Me or not, exploring a key idea for a tentative SAR. This *in vitro* enzyme inhibitory study rationalized the preliminary SAR of the parents **1**–**3** and indicated that the chemical modifications in such target compounds produced eleven derivatives **(4**–**14)** with different enzymatic inhibitory activities.

The SAR study suggested that the inhibitory activity mainly relies on the presence of aliphatic or aromatic groups at the position R_2_. However, in case of aromatic group, it relies on the type and position of its substituents. This activities changing depends on the presence of an electron withdrawing or donating groups which might be responsible for its binding with enzyme. Moreover, the presence of bulky moiety as aromatic group substituents affects on the compound affinity toward the enzyme. In the present study, the substitution pattern of the triazoloquinazoline scaffold at position 4 in parent compounds **1**–**3** induced potent activity. Thus, the compounds **4** (Ethyl) and **5** (Allyl) were more potent, with a two- and three-fold enhanced inhibitory activity ((IC_50_ = 72.28 ± 4.67 and 45.65 ±4.28 μM) compared with that of acarbose (IC_50_ = 143.54 ± 2.08 μM). This might be because the transformed targets **4** and **5** attained adequate conformation to fit the active site of α-glucosidase. Similarly, the transformation of parent **3** into **14** drastically increased its inhibitory activity, suggesting that the presence of heteroalkyl group could enhance the affinity toward α-glucosidase. Moreover, this indicated that the relative changes in triazoloquinazoline bulky structure lead to significant difference in its ability to inhibit α-glucosidase as shown by compounds **12** (N-Ethyl pyridine) and **14** (3-CN-benzyl). Notably, a sharp decrease in the inhibitory activity was observed in all compounds bearing a 4-aromatic substituent (**6, 9, 10, 11, 13**). Although, there was an increase in the lipophilicity character, the methyl group in **6, 10, 11, 13** does not display any interaction role with the active site of the glucosidase enzyme.

As mentioned in the introductory part regarding the benzoquinazolines (potent glucosidase inhibitors), we observed that such derivatives bearing electron withdrawing group substituted on benzyl group showed higher inhibitory effects. Recognizing the importance of the quinazoline moiety in several benzoquinazoline and triazoloquinazoline pharmaceutical compounds, we investigated the modifications of our targets that might contribute to improve α-glucosidase inhibitory activity. Moreover, our SAR study on benzoquinazolines validated that the increasing of the number of H-bonds formed, the binding energy of the stable complex formed between the docked benzoquinazolines, and the amino acids in the active site of the enzyme and the number of aromatic rings positively enhanced the inhibitory effect [8].

In view of the aforementioned facts, one of our aims was to incorporate such electron withdrawing substitutions in the target triazoloquinazolines **6**, **9**, **10**, **11,** and **13**. Contrarily, the inhibitory activity was abolished when parents **1** and **3** were transformed into **6**, **9**, **10, 11,** and **13.** This suggests that the insertion of aromatic substitutions with electron withdrawing groups might either behave as a deactivating group that restricts the affinity between inhibitors and enzyme or did not adequately fit the conformation of the enzyme’s active site.

### Molecular docking results

**Fig 4:**
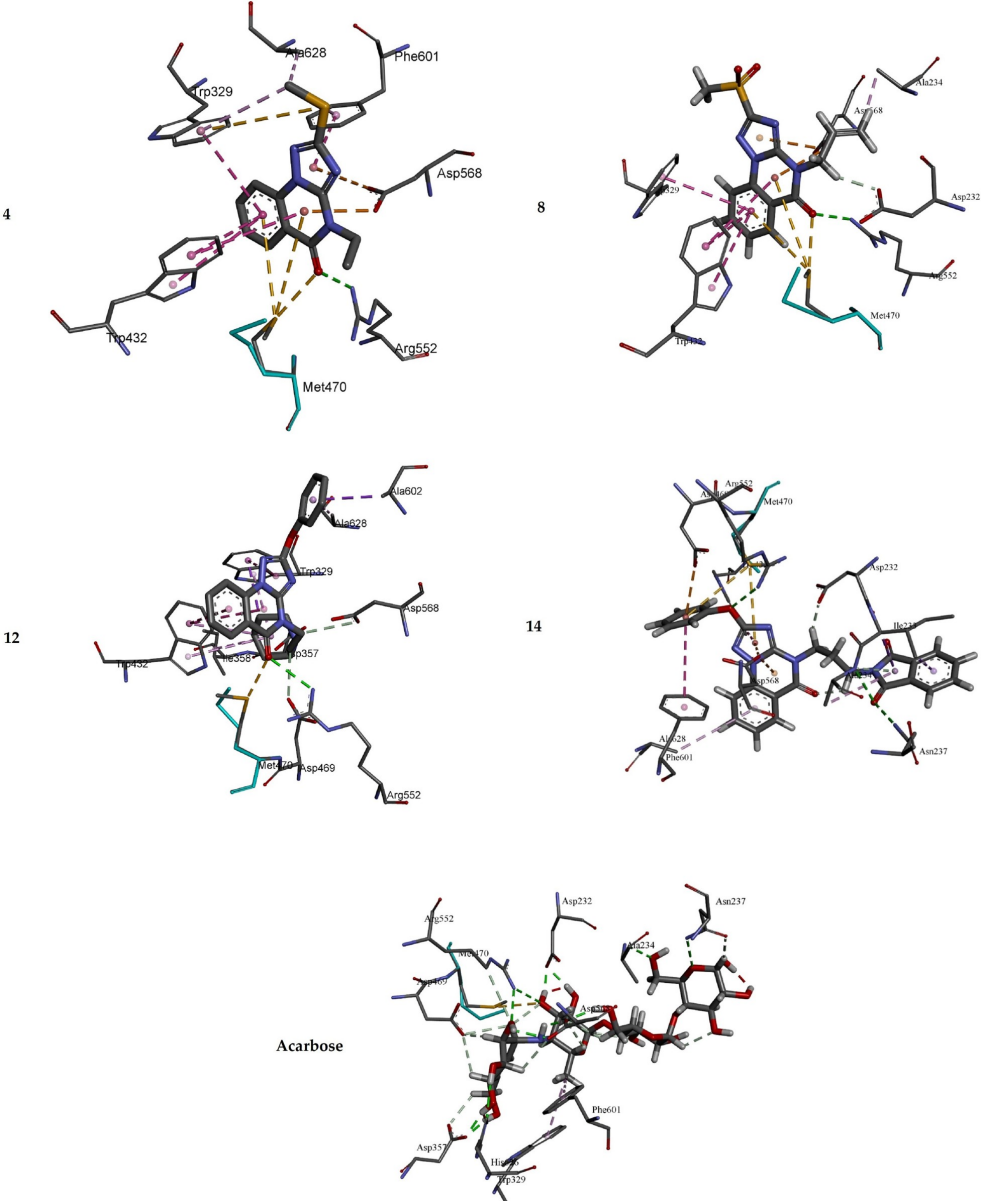
3D of the closest interactions between the active site residues of α-glucosidase and the highly active compounds 14, 4, 8, and acarbose.

**Fig 5:**
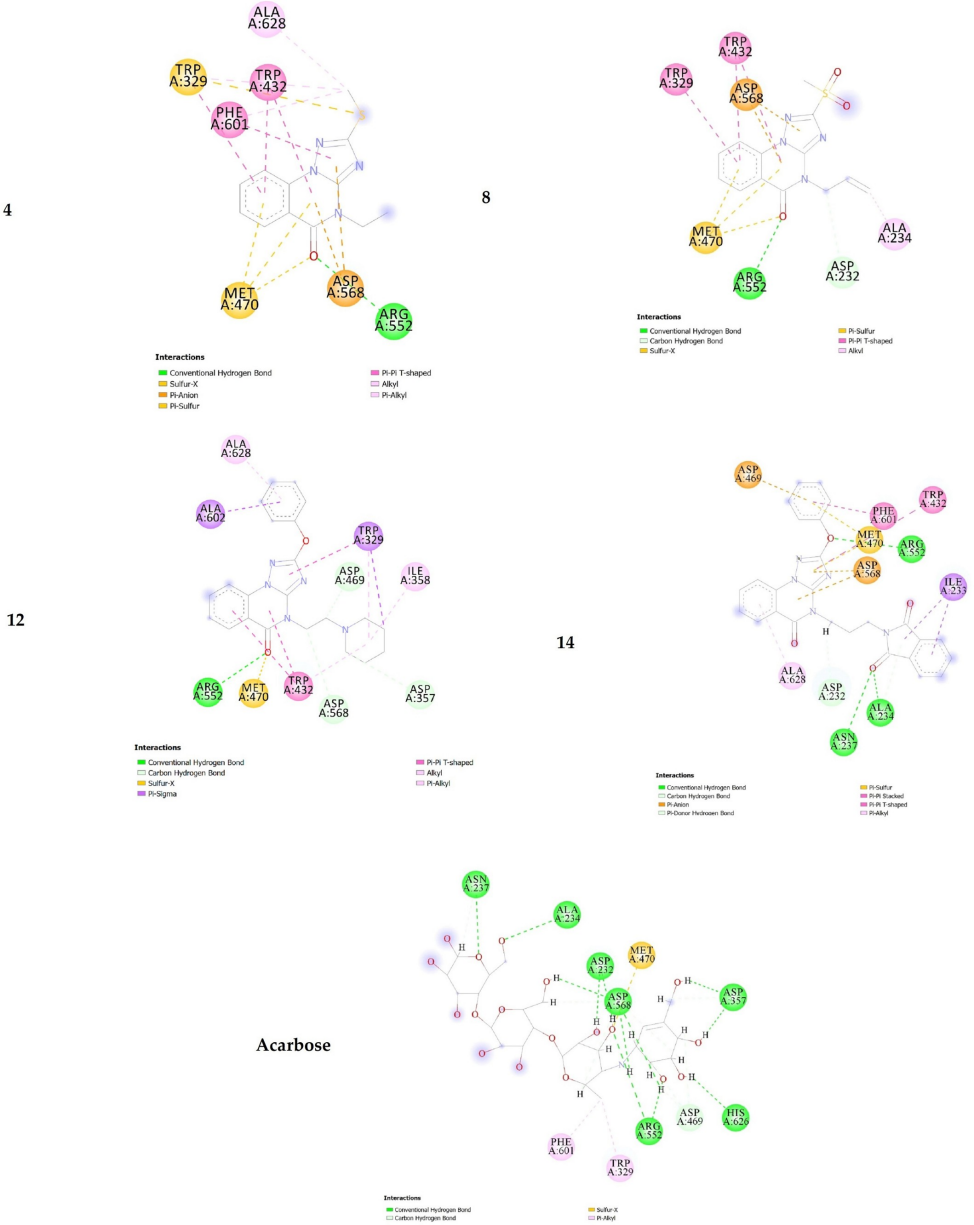
2D of the closest interactions between the active site residues of α-glucosidase and the moderately active compounds 14, 4, 8, and acarbose.

The potential antidiabetic activity of the target triazoloquinazolines was explored by evaluating their ability to inhibit α-glucosidase. The results revealed that compound **14** is the best inhibitor of α-glucosidase. In light to our previous studies using similar parameters [8], molecular docking study was carried out to elucidate the binding modes between the docked triazoloquinazolines (**4**, **8**, **12** and **14**) and α-glucosidase enzyme. The binding energy, number of hydrogen bonds, and the number of closest residues surrounding the docked triazoloquinazolines **4**, **8**, **12** and **14** into the active site of α-glucosidase were determined (Table 2 and Figs 4 and 5). All the stable complexes formed between the docked triazoloquinazolines and α-glucosidase displayed negative binding energy. This indicates that the inhibition of α-glucosidase by the tested compounds is favorable from thermodynamic point of view.

**Table 2:** Docking binding energy and the inhibition activity of the docked triazoloquinazolines into the active site of α-glucosidase.

Synthesized derivatives	Free binding energy (kcal/mol)	Number of HBs	Number of closest residues	IC_50_ (μM)
**4**	-7.28	1	7	45.56
**8**	-7.74	1	7	28.54
**12**	-11.76	1	10	97.53
**14**	-10.48	3	11	12.70
**Acarbose**	-10.56	10	10	143.54

From the results in Table 2, it is obvious that the higher activity of **14** compared with that of **4, 8** and **12** is mainly refer to the number of hydrogen bonds formed in the **14**-α-glucosidase complex. Indeed, three hydrogen bonds were formed between compound **14** and the amino acids in the active site of α-glucosidase, whilst only one hydrogen bond is formed between the docked compounds (**4**, **8** and **12**) and the amino acids into the active site of α-glucosidase. Triazoloquinazoline **14** is substituted by phenoxy group at position 2 and propyl isoindole group at position 4. Docking study results showed that compound **14** forms hydrogen bond between the oxygen atom of phenoxy group of **14** and Arg A 552 of distance 3.04 Ǻ. The second and third hydrogen bonds are established between the carbonyl group of isoindole of compound **14** and Ala A 234 and Asn A 237 of distances 2.98 and 3.02 Ǻ, respectively. However, in case of the docked **4**, **8** and **12**, only one conventional hydrogen bond was formed between the oxo group of quinazoline and the Arg A 552 of **4**, **8** and **12** of distances 2.78, 2.86 and 2.94 Ǻ, respectively. Compound **8** showed higher inhibition efficiency compared with **4**, which may refer to the stability of **8**-α-glucosidase (Table 2). Both compounds exhibit almost similar intermolecular interaction with the active amino acids of α-glucosidase (Figs 4 and 5). In **8**-α- glucosidase complex, the allyl group at position 4 of **8** forms two intermolecular interactions with ASP 232 and ALA 234 amino acids. The hydrogen atom of -CH_2_- of the allyl group of **8** forms a carbon hydrogen bond with the oxygen atom of the carbonyl group of ASP 232 of 2.15 Ǻ, which might explain the stability of complex formed with **8** compared with the one formed with **4** (Figs 4 and 5). ALA 234 of α-glucosidase forms an alkyl interaction with the hydrogen atom (= CH2) of allyl group at position 4 of **8**, whilst the ethylate position 4 in **4** showed no interactions with amino acids of alpha-glucosidase. Both compounds exhibit Pi-Pi T-Shaped interactions with quinazolin-4(1H)-one and TRP329 and TRP432 amino acids (Fig 5).

The reference acarbose showed moderate activity (IC_50_ = 143.54 μM) compared with that of the active compounds **4**, **8**, **12** and **14**. The hydrogen bonds are the main binding interaction modes between the acarbose and the amino acids at the active site (Figs 4 and 5), which maintain complex stability. Indeed, 12 hydrogen bonds are formed between the active amino acids of α-glucosidase -glucosidase. 10 of out of the 12 hydrogen bonds are established between the hydroxyl groups of acarbose and the active amino acids of α-glucosidase. The two other hydrogen bonds are formed between (i) ASP 568 and hydrogen atom of amine NH in acarbose of a distance of 1.74 Ǻ; (ii) ASN 237 and the lone pair of the oxygen atom of tetrahydro-2H-pyrane in acarbose of a distance of 2.90 Ǻ. The other non-hydrogen bonding interactions are of type carbon hydrogen bond, Pi-Alkyl and Sulfur-X interactions (Fig 5). The lower activity of the reference drug compared with **4**, **8**, **12** and **14** may refer to the fact that the core structure of the triazoloquinazoline and the additional phenoxy can participate in the formation of p-p interactions, which are not established in acarbose-alpha-glucosidase complex. For instance, in compound **14**, it is found that (i) Pi-Pi stacked interaction is formed between the phenyl ring of PHE 601 amino acid of α-glucosidase and the phenoxyl group of **14** with a distance of 5.91 Ǻ; (ii) Pi-Pi T-shaped interaction is formed between the isoindoline moiety of TRP 432 amino acid and the triazole of **14** with a distance of 5.80 Ǻ; and (iii) Pi-Donor hydrogen between the hydrogen atom of the amide of ALA 234 and Pi orbitals of isoindoline-1,3-dionyl group of **14** with a distance of 4.08 Ǻ. Moreover, p-anion interactions might also exist as a noncovalent intermolecular force between the additional phenyl groups of some of these compounds and ionized residues of amino acid. Overall, the results suggest multiple binding modes of these AGIs.

## Conclusions

The target triazoloquinazolines **1**–**14** were identified as a novel class of potential AGIs. Their structural features and various substitutions at positions 2 and 4 in the triazole and quinazoline rings apparently played crucial roles in the inhibitory activity. However, difference in the α-glucosidase inhibitory activity is conferred by the type of substituents at both positions in the triazoloquinazoline ring. The substitution pattern in the quinazoline moiety at position 4 in parent compounds **1–3** induced potent activity, with compound **3** being more potent. Triazoloquinazolines **4**, **8**, and **12** and **14** showed the highest potential inhibitory activity against α-glucosidase and indicated their potential as potent lead candidates. Furthermore, molecular modeling study confirmed the importance of binding energy in the stability of complex formed between the docked triazoloquinazolines and the amino acid residues in the active site of the enzyme. It also demonstrated considerable interaction with the active site residues, which is in agreement with the reported IC_50_ values. Thus, triazoloquinazolines might be the best candidates for designing and discovering novel AGIs, after *in vitro/in vivo* efficacy, safety, and clinical studies.

## Supporting information

S1 Fig α-Glucosidase inhibitory activity of triazoloquinazolines. (TIF)
