# Supplementary figures and images for: Triazoloquinazolines as a new class of potent α-glucosidase inhibitors: in vitro evaluation and docking study
Source: PLoS One. 2019 Aug 14;14(8):e0220379. doi: 10.1371/journal.pone.0220379 (PMC6693780; doi:10.1371/journal.pone.0220379)

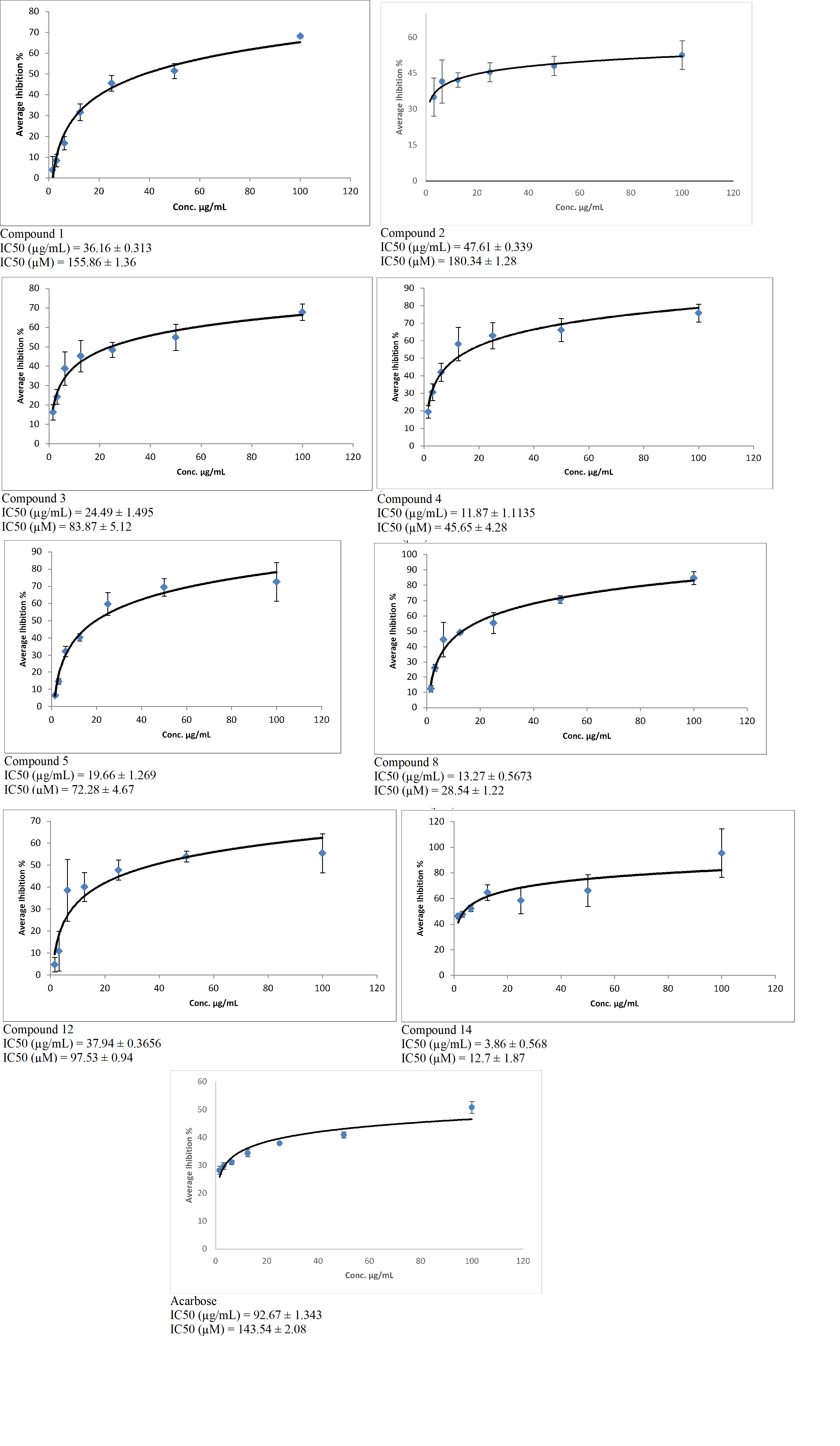

Supplement: S1 Fig — (TIF) [file pone.0220379.s001.tif]
